# Supplementary material for: Epigenetic Biomarkers of Transition from Metabolically Healthy Obesity to Metabolically Unhealthy Obesity Phenotype: A Prospective Study
Source: Int J Mol Sci. 2021 Sep 27;22(19):10417. doi: 10.3390/ijms221910417 (PMC8508854; doi:10.3390/ijms221910417)

Supplementary Table S1: Significantly differentially methylated CpG sites selected according to their contribution to the PCA.

**Cpg sites**	**Position**	**Relation**	**Gene**	**Island position**	**Location**
**cg20707527**	8 q23.1	Genetic	ZFPM2	S_Shore	
**cg20381372**	19 q13.12	Intergenic	LOC100134317	S_Shelf	TSS1500
**cg20022036**	6 p21.32	Genetic	HLA-DRB1	N_shelf	Body
**cg15084585**	8 q23.1	Genetic	ZFPM2	S_Shore	
**cg23024343**	7 q22.3	Genetic	COG5	N_Shelf	Body
**cg20485733**	17 p13.3	Genetic	RAP1GAP2	Open Sea	Body
**cg20239921**	7 q 11.23	Genetic (Pseudogene)	DTX2P1-UPK3BP1-PMS2P11	Open Sea	Body
**cg07040661**	11 p15.5	Genetic	MUC2	N_Shelf	Body
**cg25458175**	19 q13.43	Genetic	ZBTB45	Island	Body
**cg07180897**	6 p21.32	Genetic	HLA-DQB2	N_Shore	Body
**cg08720517**	5 q 31.2	Genetic	PROB1	Island	1st Exon
**cg14073541**	9 q 33.3	Genetic	DENND1A	Open Sea	Body
**cg07458466**	12 p 12.2	Genetic	PLCZ1	Open sea	Body
**cg09642739**	8 p 11.21	Genetic	HOOK3	Open Sea	Body
**cg22949274**	11 q 23.1	Genetic	SDHD	S_Shelf	Body
**cg01437515**	1 p 36.33	Genetic	TNFRSF4		
**cg24840300**	19 p13.3	Genetic	PLPR3	Island	3'UTR
**cg25828445**	12 p13.31	Intergenic (Pseudogene)	NIFKP3	S_Shore	
**cg11445109**	10 q26.3	Genetic	CYP2E1	S_Shore	Body
**cg19469447**	10 q26.3	Genetic	CYP2E1	Island	Body
**cg17172308**	6 q27	Intergenic	FRMD1	S_Shelf	
**cg05473257**	10 q26.3	Genetic	CYP2E1	Island	Body
**cg14906510**	12 p 13.31	Genetic	NIFKP3	Island	
**cg05194426**	10 q26.3	Genetic	CYP2E1	S_Shore	Body
**cg23400446**	10 q26.3	Genetic	CYP2E1	Island	Body
**cg26805839**	9p24.2	Genetic	SLC1A1	Open Sea	Body

Supplementary Table S2: Significantly differentially methylated CpG sites in the in-depth analysis of identified genes in the double PCA selection.

Gene	Cpg	P-value
ZBTB45e	cg18521743	0.0450
	cg21517584	0.0079
	cg10598434	0.0249
DENND1A	cg14073541	0.0392
RAP1GAP2	cg20485733	0.0443
	cg15586931	0.0415
	cg21022776	0.0369
MUC2	cg08804537	0.0272
	cg26740937	0.0490
	cg27483767	0.0481
	cg11415512	0.0291
Zona 5´ MUC2	cg05285917	0.0278
Zona 3´ MUC2	cg12306296	0.0244
CYP2E1	cg13315147	0.0071
	cg10862468	0.0375
	cg25330361	0.0489
	cg24530264	0.0390
	cg18984983	0.0053
HLA-DRB1	cg19575208	0.0051
	cg10765922	0.0040
	cg26036029	0.0300
	cg24147543	<0.001
ZFPM2	cg21425607	0.0138
	cg27281791	0.0197
	cg00112309	0.0484
SLC1A1	cg02464768	0.0037
	cg16545428	0.0419
DTX2P1-UPK3BP1-PMS2P11	cg12123692	0.0264
COG5	cg24382300	0.0122
	cg24648061	0.0404
	cg06593906	0.0360
	cg22438280	0.0203
HLA-DQB2	cg20645912	0.0137
	cg03638120	0.0104
	cg18609891	0.0265
PLCZ1	cg25473831	0.0183
	cg21522539	0.0448
	cg24806623	0.0466
HOOK3	cg17145652	0.0425
	cg00741600	0.0234

	cg02204844	0.0204
	cg11371500	0.0204
FRDM1	cg03002835	0.0085
DTX2P1-UPK3BP1-PMS2P11 flank	cg16669468	0.0024

Supplementary Table S3: Gene ontology annotation of the 26 differentially methylated CpG sites.

Gene Ontologyaccession (term)	CpG site	Gene
CELULAR COMPONENTS		
**GO:0032588** (trans-Golgi network membrane)	cg23024343	component of oligomericgolgi complex 5(COG5)
	cg20022036	major histocompatibility complex, class II, DR beta 1 (HLA-DRB1)
	cg07180897	major histocompatibility complex, class II, DQ beta 2 (HLA-DQB2)
**GO:0042613** (MHC class II protein complex)	cg07180897	major histocompatibility complex, class II, DQ beta 2 (HLA-DQB2)
	cg20022036	major histocompatibility complex, class II, DR beta 1 (HLA-DRB1)
**GO:0030669** (Clathrin-coated endocytic vesicle membrane)	cg20022036	major histocompatibility complex, class II, DR beta 1 (HLA-DRB1)
	cg07180897	major histocompatibility complex, class II, DQ beta 2 (HLA-DQB2)
**GO:0042611** (MHC class protein complex)	cg07180897	major histocompatibility complex, class II, DQ beta 2 (HLA-DQB2)
	cg20022036	major histocompatibility complex, class II, DR beta 1 (HLA-DRB1)
**GO:0071556** (Integral component of luminal side of endoplasmic reticulum membrane)	cg20022036	major histocompatibility complex, class II, DR beta 1 (HLA-DRB1)
	cg07180897	major histocompatibility complex, class II, DQ beta 2 (HLA-DQB2)
**GO:0098553** (lumenal side of endoplasmic reticulum membrane)	cg20022036	major histocompatibility complex, class II, DR beta 1 (HLA-DRB1)
	cg07180897	major histocompatibility complex, class II, DQ beta 2 (HLA-DQB2)
**GO:0031227** (intrinsic component of endoplasmic reticulum membrane)	cg20022036	major histocompatibility complex, class II, DR beta 1 (HLA-DRB1)
	cg07180897	major histocompatibility complex, class II, DQ beta 2 (HLA-DQB2)
**GO:0098576** (lumenal side of membrane)	cg20022036	major histocompatibility complex, class II, DR beta 1 (HLA-DRB1)
	cg07180897	major histocompatibility complex, class II, DQ beta 2 (HLA-DQB2)
**GO:0030662** (coated vesicle membrane)	cg20022036	major histocompatibility complex, class II, DR beta 1 (HLA-DRB1)
	cg07180897	major histocompatibility complex, class II, DQ beta 2 (HLA-DQB2)
MOLECULAR FUNCTION		
**GO:0032395**	cg20022036	major histocompatibility complex, class II, DR beta 1 (HLA-DRB1)

(MHC class II receptor activity)	cg07180897	major histocompatibility complex, class II, DQ beta 2 (HLA-DQB2)
**GO:0033229** (cysteine transmembrane transporter activity)	cg26805839	solute carrier family 1 member 1 (SLC1A1)
**GO:0140010** (D-aspartate transmembrane transporter activity)	cg26805839	solute carrier family 1 member 1 (SLC1A1)
**GO:0018601** (4-nitrophenol 2-monooxygenase activity)	cg05473257	Cytochrome P450 2E1(CYP2E1)
	cg05194426	
	cg23400446	
	cg11445109	
	cg19469447	
**GO:0032266** (phosphatidylinositol-3-phosphate binding)	cg07458466	phospholipase C, zeta 1 (PLCZ1)
**GO:0042943** (D-amino acid transmembrane transporter activity)	cg26805839	solute carrier family 1 member 1 (SLC1A1)
**GO:0000104** (succinate dehydrogenase activity)	cg22949274	Succinate dehydrogenase [ubiquinone] (SHDH)
**GO:0140375** (immune receptor activity)	cg20022036	major histocompatibility complex, class II, DR beta 1 (HLA-DRB1)
	cg07180897	major histocompatibility complex, class II, DQ beta 2 (HLA-DQB2)
**GO:0020037** (heme binding)	cg22949274	Succinate dehydrogenase [ubiquinone] (SHDH)
**GO:0005314** (high-affinity glutamate transmembrane transporter activity)	cg26805839	solute carrier family 1 member 1 (SLC1A1)
BIOLOGICAL PROCESS		
**GO:0060470** (positive regulation of cytosolic calcium ion concentration involved in egg activation)	cg07458466	phospholipase C, zeta 1 (PLCZ1)
**GO:1903712** (cysteine transmembrane transport)	cg26805839	solute carrier family 1 member 1 (SLC1A1)

**GO:0018885**<br>(carbon tetrachloride metabolic process)	cg05473257<br>cg05194426<br>cg23400446<br>cg11445109<br>cg19469447	Cytochrome P450 2E1(CYP2E1)
**GO:0018910**<br>(benzene metabolic process)	cg05473257<br>cg05194426<br>cg23400446<br>cg11445109<br>cg19469447	Cytochrome P450 2E1(CYP2E1)
**GO:0018960**<br>(4-nitrophenol metabolic process)	cg05473257<br>cg05194426<br>cg23400446<br>cg11445109<br>cg19469447	Cytochrome P450 2E1(CYP2E1)
**GO:0048219**<br>(inter-Golgi cisterna vesicle-mediated transport)	cg23024343	Golgi complex subunit 5 (COG5)
**GO:0042883**<br>(cysteine transport)	cg26805839	solute carrier family 1 member 1 (SL1CA1)
**GO:0042197**<br>(halogenated hydrocarbon metabolic process)	cg05473257<br>cg05194426<br>cg23400446<br>cg11445109<br>cg19469447	Cytochrome P450 2E1(CYP2E1)
**GO:0016098**<br>(monoterpenoid metabolic process)	cg05473257<br>cg05194426<br>cg23400446<br>cg11445109<br>cg19469447	Cytochrome P450 2E1(CYP2E1)

**GO:0006121**<br>(mitochondrial electron transport, succinate to ubiquinone)	cg22949274	Succinate dehydrogenase [ubiquinone] (SHDH)

Supplementary Figure S1. Top 10 Gene Ontology (GO) terms enriched by differentially methylated genes in stable MHO and unstable MHO groups. Enriched GO terms were categorized into (**a**) biological processes, (**b**) molecular function and (**c**) cellular components. Data are presented as enriched scores expressed as -log10 (p value).

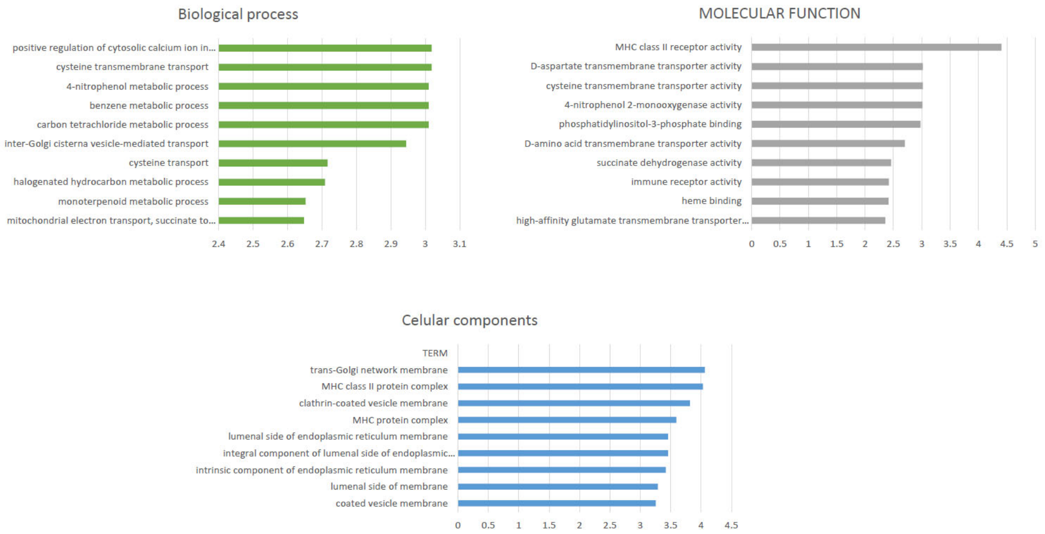

Supplement: Supplementary file 1 [file ijms-22-10417-s001.zip › ijms-1380600-supplementary.pdf]
